# Daily Steps Buffer the Impact of Daily Stress on Mood in Youth and Middle-aged/Older Adults

**DOI:** 10.1093/geroni/igab046.3071

**Published:** 2021-12-17

**Authors:** Daniel Fleming, Yin Liu, Myles Maxey, Elizabeth Braungart Fauth, Troy Beckert

**Affiliations:** 1 Utah State University, Logan, Utah, United States; 2 Utah State University, Utah State University, Utah, United States

## Abstract

Physical activity has known associations with lower stress and improved well-being. These studies often include samples from one developmental phase at a time, which is helpful for researchers in those developmental areas, but less informative for identifying predictors of health and well-being across the lifespan. The current study examined whether protective aspects of physical activity (steps) on stress and mood worked similarly in widely different age cohorts. We also examined these relationships at the daily level, as opposed to global/macro levels. Participants (n = 119, 67% female) were 44 adolescents between 13-18 years (Mage (SD) = 15.73 (1.48) years, 57% female) and 77 middle-aged/older adults between 55-76 years (Mage (4.97) = 59.67, 74% female). They self-reported global life satisfaction and demographic characteristics at baseline and completed ecological momentary assessments (three per day for three consecutive days, across six measurement bursts, each spaced two weeks apart) via smart phones, reporting on their mood, stressor exposures/types, and end-of-day pedometer step count. Multilevel models showed that daily steps had protective effects against social network stressors on both daily mood and life satisfaction, such that more steps weakened the negative relationship between network-related stressors, mood, and life satisfaction. This protective effect was uniform for both older and younger adults, and across boys/men and girls/women. Overall, the present study suggested the importance of physical activity, even that of general step count, on buffering daily stress on daily mood and general life satisfaction for participants at multiple phases of the lifespan.

